# Supplementary material for: A UMLS-based spell checker for natural language processing in vaccine safety
Source: BMC Med Inform Decis Mak. 2007 Feb 12;7:3. doi: 10.1186/1472-6947-7-3 (PMC1805499; doi:10.1186/1472-6947-7-3)
Supplement: Additional file 13 — RAP application directory. Sets up directory for RAP (RDF application for PHP) source code [file 1472-6947-7-3-S13.gz › rap/doc/phpdoc/__filesource/fsource_infModel__infModelInfModelB.php.html]

xml version="1.0" encoding="iso-8859-1"?


File Source for InfModelB.php


# Source for file InfModelB.php

Documentation is available at InfModelB.php

```
1. <?php
2. // ----------------------------------------------------------------------------------
3. // Class: InfModelB
4. // ----------------------------------------------------------------------------------


8. /**
9. * A InfModelB extends the InfModel Class, with a backward chaining algorithm.
10. * Only the loaded or added base-triples are stored.
11. * A find-query evaluates the inference rules and recursively tries to find the statements.
12. * InfModelB memorises "Dead-Ends" until the next add() command, thus
13. * makin a second find much faster.
14. * InfModelB is safe for loops in Ontologies, that would cause infinite loops.
15. * WARNING: A find(null,null,null) might take very long.
16. *
17. * <BR><BR>History:<UL>
18. * <LI>10-07-2004                 : Function findFirstMatchingStatement() added paramter $offset
19. *                                 to set an search offset
20. * <LI>09-10-2004                : First version of this class.</LI>
21. * <LI>09-15-2004                : Added Index over InRule Entailments
22. *                                  to increase performance
23. *</UL>
24. * @version  V0.9.1
25. * @author Daniel Westphal <mail at d-westphal dot de>
26. *
27. * @package infModel
28. * @access    public
29. ***/

31. class InfModelB extends InfModel
32. {

34. /**
35. * Array that holds combinations of inference rules with distinct
36. * find-querys, that don't lead to any inference.
37. * @var        array
38. * @access    private
39. */
40. var $findDeadEnds;


43. /**
44. * Constructor
45. * You can supply a base_uri
46. *
47. * @param string $baseURI
48. * @access    public
49. */
50. function InfModelB($baseURI = null)
51. {
52. parent::InfModel($baseURI);
53. $this->findDeadEnds=array();
54. }

56. /**
57. * Adds a new triple to the Model without checking, if the statement
58. * is already in the Model. So if you want a duplicate free Model use
59. * the addWithoutDuplicates() function (which is slower then add())
60. *
61. * @param    object Statement    $statement
62. * @access    public
63. * @throws    PhpError
64. */
65. function add($statement)
66. {
67. parent::add($statement);
68. //Reset the found dead-ends.
69. $this->findDeadEnds=array();
70. }

72. /**
73. * General method to search for triples.
74. * NULL input for any parameter will match anything.
75. * Example:  $result = $m->find( NULL, NULL, $node );
76. * Finds all triples with $node as object.
77. * Returns an empty MemModel if nothing is found.
78. * To improve the search speed with big Models, call index(INDEX_TYPE)
79. * before seaching.
80. *
81. * It recursively searches in the statements and rules to find
82. * matching statements
83. *
84. * @param    object Node    $subject
85. * @param    object Node    $predicate
86. * @param    object Node    $object
87. * @return    object MemModel
88. * @access    public
89. * @throws    PhpError
90. */
91. function find($subject,$predicate,$object)
92. {
93. $searchStringIndex=array();
94. $resultModel=new MemModel();

96. //add all infered statements without duplicates to the result model
97. foreach ($this->_infFind($subject,$predicate,$object,array())as $statement)
98. {
99. $resultModel->addWithoutDuplicates($statement);
100. };
101. return $resultModel;
102. }

104. /**
105. * This is the main inference method of the InfModelB
106. * The algorithm works as follows:
107. * Find all statements in the base model, that matches the current
108. * find-query.
109. * Check all rules, if they are able to deliver infered statements,
110. * that match the current find-query. Don't use rules with queries,
111. * that lead to dead-ends and don't use a rule-query-combination that
112. * was used before in this branch (ontology loops).
113. * If a rule is possible do deliver such statements, get a new
114. * find-query, that is possible to find those statements, that are able
115. * to trigger this rule.
116. * Call this _infFind method wirh the new find-query and entail the
117. * resulting statements.
118. * If this rule, wasn't able to return any statements with this distinct
119. * query, add this combination to the dead-ends.
120. * Return the statements from the base triples and those, which were infered.
121. *
122. * If $findOnlyFirstMatching is set to true, only the first match in
123. * the base-statements is entailed an returned (used in contains() and
124. * findFirstMatchingStatement() methods).
125. *
126. * You can set an offset to look for the first matching statement by setting the
127. * $offset var.
128. *
129. * It recursively searches in the statements and rules to find matching
130. * statements
131. *
132. * @param    object Node    $subject
133. * @param    object Node    $predicate
134. * @param    object Node    $object
135. * @param    array        $searchStringIndex
136. * @param    boolean     $findOnlyFirstMatching
137. * @param    integer     $offset
138. * @param    integer     $resultCount
139. * @return    object array Statements
140. * @access    private
141. */
142. function _infFind ($subject,$predicate,$object, $searchStringIndex, $findOnlyFirstMatching = false, $offset = 0,$resultCount = 0 )
143. {
144. $return=array();
145. //Find all matching statements in the base statements
146. $findResult=parent::find($subject,$predicate,$object);
147. //For all found statements
148. foreach ($findResult->triples as $statement)
149. {
150. $return[]=$statement;
151. $resultCount++;

153. //Return, if only the firstMatchingStatement was wanted
154. if ($findOnlyFirstMatching && $resultCount > $offset)
155. return $return;
156. };

158. //Don't infer statements about the schema (rdfs:subClass, etc..)
159. //is false
160. if ($predicate == null ||
161. (is_a($predicate,'Node') &&
162. !in_array($predicate->getLabel(),$this->supportedInference))
163. )
164. //Check only Rules, that the EntailmentIndex returned.
165. foreach ($this->_findRuleEntailmentInIndex($subject,$predicate,$object) as $ruleKey)
166. {
167. $infRule=$this->infRules[$ruleKey];
168. $serializedRuleStatement=$ruleKey.serialize($subject).serialize($predicate).serialize($object);
169. //If it is to ontology loop and no dead-end
170. if (!in_array($serializedRuleStatement, $searchStringIndex) &&
171. !in_array($serializedRuleStatement, $this->findDeadEnds))
172. {
173. //Keep this distinct rule query cobination for
174. //this branch to detect loops
175. $searchStringIndex[]=$serializedRuleStatement;

177. //If the rule is able to deliver statements that match
178. //this query
179. if ($infRule->checkEntailment($subject,$predicate,$object))
180. {
181. //Get a modified find-query, that matches statements,
182. //that trigger this rule
183. $modefiedFind=$infRule->getModifiedFind($subject,$predicate,$object);
184. //Call this method with the new find-query
185. $infFindResult=$this->_infFind($modefiedFind['s'],
186. $modefiedFind['p'],
187. $modefiedFind['o'],
188. $searchStringIndex,
189. $findOnlyFirstMatching,
190. $offset,
191. $resultCount) ;
192. //If it deliverd statements that matches the trigger
193. if (isset($infFindResult[0]))
194. {
195. foreach ($infFindResult as $statement)
196. {
197. //Entail the statements and check, if they are not about the
198. //ontology
199. $newStatement=$infRule->entail($statement);
200. if (!in_array($newStatement->getLabelPredicate(),$this->supportedInference))
201. //Check if, the entailed statements are, what we are looking for
202. if($this->_nodeEqualsFind($subject,$newStatement->getSubject()) &&
203. $this->_nodeEqualsFind($predicate,$newStatement->getPredicate()) &&
204. $this->_nodeEqualsFind($object,$newStatement->getObject() ) )
205. {
206. //Add to results
207. $return[]=$newStatement;
208. $resultCount++;

210. //or return at once
211. if ($findOnlyFirstMatching && $resultCount > $offset)
212. return $return;
213. }
214. }
215. } else
216. {
217. //If there were no results of the rule-query-combination,
218. //mark this combination as a dead-end.
219. $this->findDeadEnds[]=$serializedRuleStatement;
220. }
221. }
222. }
223. }
224. //Return the array of the found statements
225. return $return;
226. }

228. /**
229. * Tests if the Model contains the given triple.
230. * TRUE if the triple belongs to the Model;
231. * FALSE otherwise.
232. *
233. * @param    object Statement    &$statement
234. * @return    boolean
235. * @access    public
236. */
237. function contains(&$statement)
238. {
239. //throws an error, if $statement is not of class Statement
240. if(!is_a($statement,'Statement'))
241. trigger_error(RDFAPI_ERROR . '(class: InfModelB; method: contains):
242. $statement has to be object of class Statement', E_USER_ERROR);

244. //Call the _infFind method, but let it stop, if it finds the first match.
245. if (count( $this->_infFind($statement->getSubject(),
246. $statement->getPredicate(),
247. $statement->getObject(),
248. array(),true) ) >0)
249. {
250. return true;
251. } else
252. {
253. return false;
254. };
255. }

257. /**
258. * Searches for triples and returns the first matching statement.
259. * NULL input for any parameter will match anything.
260. * Example:  $result = $m->findFirstMatchingStatement( NULL, NULL, $node );
261. * Returns the first statement of the MemModel where the object equals $node.
262. * Returns an NULL if nothing is found.
263. * You can define an offset to search for. Default = 0
264. *
265. * @param    object Node    $subject
266. * @param    object Node    $predicate
267. * @param    object Node    $object
268. * @param    integer    $offset
269. * @return    object Statement
270. * @access    public
271. */
272. function findFirstMatchingStatement($subject, $predicate, $object, $offset = 0)
273. {
274. //Call the _infFind method, but let it stop, if it finds the
275. //first match.
276. $res= $this->_infFind($subject,$predicate,$object,array(),true,$offset);

278. if (isset($res[$offset]))
279. {
280. return $res[$offset];
281. } else
282. {
283. return NULL;
284. };
285. }

287. /**
288. * Returns a StatementIterator for traversing the Model.
289. * @access    public
290. * @return    object    StatementIterator
291. */
292. function & getStatementIterator()
293. {
294. // Import Package Utility
295. include_once(RDFAPI_INCLUDE_DIR.PACKAGE_UTILITY);
296. // Gets a MemModel by executing a find(null,null,null) to get a
297. //inferable statements.
298. // WARNING: might be slow
299. return new StatementIterator($this->getMemModel());
300. }

302. /**
303. * Number of all inferable triples in the Model.
304. * WARNING: uses a find(null,null,null) to find all statements! (might take a while)
305. *
306. * @param     boolean
307. * @return    integer
308. * @access    public
309. */
310. function size()
311. {
312. // Gets a MemModel by executing a find(null,null,null) to get a
313. //inferable statements.
314. // WARNING: might be slow
315. $res = $this->getMemModel();
316. return $res->size();
317. }

319. /**
320. * Create a MemModel containing all the triples (including inferred
321. * statements) of the current InfModelB
322. *
323. * @return object MemModel
324. * @access public
325. */
326. function & getMemModel()
327. {

329. $return=$this->find(null,null,null);
330. $return->setBaseURI($this->baseURI);
331. $return->addParsedNamespaces($this->getParsedNamespaces());
332. return $return;
333. }

335. /**
336. * Create a MemModel containing only the base triples (without inferred
337. * statements) of the current InfModelB
338. *
339. * @return object MemModel
340. * @access public
341. */
342. function & getBaseMemModel()
343. {
344. $return= new MemModel();
345. $return->setBaseURI($this->baseURI);
346. foreach ($this->triples as $statement)
347. $return->add($statement);

349. $retun->addParsedNamespaces($this->getParsedNamespaces());
350. return $return;
351. }

353. /**
354. * Short Dump of the  InfModelB.
355. *
356. * @access    public
357. * @return    string
358. */
359. function toString()
360. {
361. return 'InfModelB[baseURI=' . $this->getBaseURI() . ';  size=' . $this->size(true) . ']';
362. }

364. /**
365. * Dumps of the InfModelB including ALL inferable triples.
366. *
367. * @access    public
368. * @return    string
369. */
370. function toStringIncludingTriples()
371. {
372. $dump = $this->toString() . chr(13);
373. $stateIt=new StatementIterator($this->find(null,null,null));
374. while($statement=$stateIt->next())
375. {
376. $dump .= $statement->toString() . chr(13);
377. }
378. return $dump;
379. }

381. /**
382. * Saves the RDF,N3 or N-Triple serialization of the full InfModelB
383. * (including inferred triples) to a file.
384. * You can decide to which format the model should be serialized by
385. * using a corresponding suffix-string as $type parameter. If no $type
386. * parameter is placed this method will serialize the model to XML/RDF
387. * format.
388. * Returns FALSE if the InfModelB couldn't be saved to the file.
389. *
390. * @access    public
391. * @param     string     $filename
392. * @param     string     $type
393. * @throw   PhpError
394. * @return    boolean
395. */
396. function saveAs($filename, $type ='rdf')
397. {

399. $memmodel=$this->getMemModel();
400. return $memmodel->saveAs($filename, $type);
401. }

403. /**
404. * Writes the RDF serialization of the Model including ALL inferable
405. * triples as HTML.
406. *
407. * @access    public
408. */
409. function writeAsHtml()
410. {
411. require_once(RDFAPI_INCLUDE_DIR.PACKAGE_SYNTAX_RDF);
412. $ser = new RdfSerializer();
413. $rdf =& $ser->serialize($this->getMemModel());
414. $rdf = htmlspecialchars($rdf, ENT_QUOTES);
415. $rdf = str_replace(' ', '&nbsp;', $rdf);
416. $rdf = nl2br($rdf);
417. echo $rdf;
418. }

420. /**
421. * Writes the RDF serialization of the Model including ALL inferable
422. * triples as HTML table.
423. *
424. * @access    public
425. */
426. function writeAsHtmlTable()
427. {
428. // Import Package Utility
429. include_once(RDFAPI_INCLUDE_DIR.PACKAGE_UTILITY);
430. RDFUtil::writeHTMLTable($this->getMemModel());
431. }


434. /**
435. * Writes the RDF serialization of the Model including ALL inferable
436. * triples.
437. *
438. * @access    public
439. * @return    string
440. */
441. function writeRdfToString()
442. {
443. // Import Package Syntax
444. include_once(RDFAPI_INCLUDE_DIR.PACKAGE_SYNTAX_RDF);
445. $ser = new RdfSerializer();
446. $rdf =& $ser->serialize($this->getMemModel());
447. return $rdf;
448. }

450. /**
451. * Removes the triple from the MemModel.
452. * TRUE if the triple is removed.
453. * FALSE otherwise.
454. *
455. * Checks, if it touches any statements, that added inference rules
456. * to the model
457. *
458. * @param    object Statement    $statement
459. * @return   boolean
460. * @access    public
461. * @throws    PhpError
462. */
463. function remove($statement)
464. {
465. if (parent::contains($statement))
466. {
467. if (in_array($statement->getLabelPredicate(),$this->supportedInference));
468. while (count($this->_removeFromInference($statement))>0);

470. $this->findDeadEnds=array();
471. return parent::remove($statement);
472. } else
473. {
474. return false;
475. }
476. }

478. /**
479. * Checks, if a single node matches a single find pattern.
480. * TRUE if the node matches.
481. * FALSE otherwise.
482. *
483. * Checks, if it touches any statements, that added inference rules
484. * to the model
485. *
486. * @param    object Statement    $statement
487. * @return   boolean
488. * @access    private
489. */
490. function _nodeEqualsFind(& $find, $node)
491. {
492. //If the find pattern is a node, use the nodes equal-method and
493. //return the result.
494. if (is_a($find,'Node'))
495. return $node->equals($find);

497. //Null-pattern matches anything.
498. if ($find == null)
499. {
500. return true;
501. } else
502. {
503. return false;
504. }
505. }

507. /**
508. * Returns a FindIterator for traversing the MemModel.
509. * Disabled in InfModelB
510. * @access    public
511. * @return    object    FindIterator
512. */
513. function & findAsIterator($sub=null,$pred=null,$obj=null) {
514. $errmsg = RDFAPI_ERROR . '(class: InfModelB; method: findAsIterator):
515. This function is disabled in the
516. Inference Model';
517. trigger_error($errmsg, E_USER_ERROR);
518. }
519. }
520. ?>
```

Documentation generated on Thu, 7 Jul 2005 13:41:38 +0200 by phpDocumentor 1.3.0RC3
